# Reviewer acknowledgement 2015

**DOI:** 10.1186/s12936-016-1211-z

**Published:** 2016-03-30

**Authors:** Marcel Hommel

**Affiliations:** 236 Grays Inn Road, London, WC1X 8HB UK

*Malaria Journal* would like to thank everyone who reviewed for the journal in 2015.

**Tarekegn Abeku**

UK

**Angela Acosta**

USA

**Ahmed Adedeji**

UK

**Martin Adjuik**

Ghana

**Yeya Adoke**

Uganda

**Hippolyte Affognon**

Kenya

**Niklas Ahlborg**

Sweden

**Michael Aidoo**

USA

**Jane Alaii**

Kenya

**Pietro Alano**

Italy

**Kassahun Alemu**

Ethiopia

**Neil Alexander**

UK

**Michael Alifrangis**

Denmark

**Jean-Pierre Allain**

UK

**Stephen Allen**

UK

**Haoues Alout**

USA

**Borko Amulic**

Germany

**Chiara Andolina**

Thailand

**Ehimen Aneni**

USA

**Brian Angus**

UK

**Evelyn Ansah**

Ghana

**Nicholas Anstey**

Australia

**Bruno Arca**

Italy

**Maru Aregawi**

Switzerland

**Paolo Arese**

Italy

**Gabriela Arevalo-Pinzon**

Colombia

**Frederic Ariey**

France

**Emmanuel Arinaitwe**

Uganda

**Benjamin Arnold**

USA

**Elizabeth Ashley**

Thailand

**Jo-Ann Atkinson**

Australia

**Sarah Auburn**

Australia

**Alyson Auliff**

Australia

**Tin Aung**

Myanmar

**Vicky Avery**

Australia

**Gordon Awandare**

Ghana

**Francesca Aweeka**

USA

**Diego Ayala**

France

**Berit Aydin-Schmidt**

Sweden

**Anna Babakhanyan**

USA

**Anna Bachmann**

Germany

**David Bacon**

USA

**John Kevin Baird**

Indonesia

**David Baker**

UK

**Kristin Banek**

UK

**Bridget Barber**

Australia

**Alyssa Barry**

Australia

**Leonardo Basco**

France

**Nicoletta Basilico**

Italy

**Quique Bassat**

Mozambique

**Imelda Bates**

UK

**Katherine Battle**

UK

**Vincent Batwala**

Uganda

**Jake Baum**

UK

**Nabie Bayoh**

Kenya

**Raymond Beach**

USA

**Katja Becker**

Germany

**Norbert Becker**

Germany

**Mohammad Beg**

Pakistan

**Ron Behrens**

UK

**John Beier**

USA

**Claudia Beiersmann**

Germany

**David Bell**

Switzerland

**Nicole Isabelle Melanie Berens-Riha**

Germany

**Aase Berg**

Norway

**Elke Bergmann-Leitner**

USA

**Pedro Berzosa**

Spain

**Nora Besansky**

USA

**Prasad Bharatam**

India

**Samir Bhatt**

UK

**Achuyt Bhattarai**

USA

**Peter Billingsley**

USA

**Lyn-Marie Birkholtz**

South Africa

**Donal Bisanzio**

UK

**Zeno Bisoffi**

Italy

**Sukla Biswas**

India

**Anders Bjorkman**

Sweden

**Andrew Blagborough**

UK

**Moses Bockarie**

UK

**Christophe Boete**

France

**Andrea Boggild**

Canada

**Michael Boivin**

USA

**Angelica Boldt**

Germany

**Arne Bomblies**

USA

**Kimberly Bonner**

Tanzania

**Isabelle Borghini-Fuhrer**

Switzerland

**Samuel Bosomprah**

UK

**Teun Bousema**

UK

**Konstantina Boutsika**

Switzerland

**Sébastien Boyer**

Madagascar

**Bernard Brabin**

UK

**John Bradley**

UK

**Paula Brentlinger**

USA

**William Brieger**

USA

**Olivier Briët**

Switzerland

**Cristiana Brito**

Brazil

**Miguel Brito**

Portugal

**Sumudu Britton**

Australia

**William Brogdon**

USA

**Basil Brooke**

South Africa

**Simon Brooker**

UK

**Iris Bruchhaus**

Germany

**Pierre Buffet**

France

**Hasifa Bukirwa**

Uganda

**Peter Bull**

UK

**Susan Bull**

UK

**Kristen Bullard**

USA

**Tom Burkot**

Australia

**Austin Burt**

UK

**M. Bustos**

Philippines

**Pauline Byakika-Kibwika**

Uganda

**Ioav Cabantchik**

Israel

**Brice Campo**

Switzerland

**Sara Canavati**

Cambodia

**Margareth Capurro**

Brazil

**Beniamino Caputo**

Italy

**Jaime Carmona-Fonseca**

Colombia

**Verena Carrara**

Thailand

**Tamar Carter**

USA

**Luzia Carvalho**

Brazil

**Francesco Castelli**

Italy

**Marcia Castro**

USA

**Hugo Castro-Faria-Neto**

Brazil

**Carla Cerami Hand**

USA

**Crispim Cerutti**

Brazil

**Carlos Chaccour**

Spain

**Debopam Chakrabarti**

USA

**Chandan Chakraborty**

India

**Emmanuel Chanda**

Zambia

**Jacques Derek Charlwood**

UK

**Susan Charman**

Australia

**Virander Chauhan**

India

**Jean-Marc Chavatte**

Singapore

**Arnaud Chene**

France

**Stella Chenet**

USA

**Qin Cheng**

Australia

**Fei Wen Cheong**

Malaysia

**Matthew Chico**

UK

**Junhong Ch’ng**

Sweden

**Palang Chotsiri**

Thailand

**Cindy Chu**

Thailand

**Antoine Claessens**

UK

**Carla Claser**

Singapore

**Justin Cohen**

USA

**Valery Combes**

Australia

**Jan Conn**

USA

**Stephen Connor**

USA

**Melissa Conrad**

USA

**Edi Constant**

Côte D’ivoire

**David Conway**

UK

**Jackie Cook**

UK

**Cheryl Cooper**

USA

**Roland Cooper**

USA

**Marc Henri Carl Coosemans**

Belgium

**Sylvie Cornelie**

France

**Stephan Cornet**

France

**Giampetro Corradin**

Switzerland

**Carlo Costantini**

France

**Michel Cot**

France

**Chris Cotter**

USA

**Mamadou Coulibaly**

Mali

**Helen Counihan**

UK

**David Courtin**

France

**Janet Cox-Singh**

UK

**Alister Craig**

UK

**Jakob Peter Cramer**

Germany

**Jane Crawley**

UK

**Peter Crompton**

USA

**Cécile Crosnier**

UK

**Liwang Cui**

USA

**Richard Culleton**

Japan

**Monica Da Silva-Nunes**

Brazil

**Peter Dambach**

Germany

**Aditya Dash**

India

**Miles Davenport**

Australia

**Nicholas Day**

Thailand

**Tania de Koning-Ward**

Australia

**Quirijn De Mast**

Netherlands

**Caroline de Oliveira**

Brazil

**J. Brian De Souza**

UK

**Eric Deharo**

Peru

**Kirk Deitsch**

USA

**Hernando A. Del Portillo**

Spain

**Alessandra Della Torre**

Italy

**Stephanie Dellicour**

UK

**Philippe Deloron**

France

**Michael Delves**

UK

**Bruna Demari-Silva**

Brazil

**Dominic Dery**

Ghana

**Sunil Dhiman**

India

**Mehul Dhorda**

Thailand

**Thierry Diagana**

Singapore

**Natalie Dial**

USA

**Maria Diuk-Wasser**

USA

**Abdoulaye Djimde**

Mali

**Luc Djogbénou**

Benin

**Christian Doerig**

Australia

**Gonzalo J. Domingo**

USA

**Arjen M. Dondorp**

Thailand

**Stefan Dongus**

Switzerland

**Cristina Donini**

Switzerland

**Martin Donnelly**

UK

**Ogobara Doumbo**

Mali

**Chris Drakeley**

UK

**Mohan Lal Dubey**

India

**Patrick Duffy**

USA

**Sandra Duffy**

Australia

**Stephen Duke**

USA

**Stephan Duparc**

Switzerland

**Lies Durnez**

Belgium

**Sheetij Dutta**

USA

**Linda Duval**

France

**Philip Eckhoff**

USA

**Michael Edstein**

Australia

**Geoffrey Edwards**

UK

**Timothy Egan**

South Africa

**Thomas Eisele**

USA

**Iqbal Elyazar**

Indonesia

**Noriko Endo**

USA

**Ananias Escalante**

USA

**Johan Esterhuizen**

UK

**Chi Eziefula**

UK

**Catherine Falade**

Nigeria

**Thierry Fandeur**

Gabon

**Caterina Fanello**

UK

**Anna Färnert**

Sweden

**Jean-François Faucher**

France

**Michael Faulde**

Germany

**Ingrid Felger**

Switzerland

**Rolf Fendel**

Germany

**Delmiro Fernandez-Reyes**

UK

**Marcelo Ferreira**

Brazil

**Pedro Ferreira**

Japan

**Santiago Ferrer**

Spain

**David Fidock**

USA

**Scott Filler**

USA

**Ulrike Fillinger**

UK

**Lawrence Fleckenstein**

USA

**Jennifer Flegg**

UK

**Gustavo Fontecha**

Honduras

**Cor Fontes**

Brazil

**Christen Fornadel**

USA

**Woodbridge Foster**

USA

**Freya Fowkes**

Australia

**Brian Foy**

USA

**Ana Franco**

Portugal

**Ariey Frederic**

Gabon

**Hagen Frickmann**

Germany

**Friedrich Frischknecht**

Germany

**Megan Fritz**

USA

**Andrew Fry**

UK

**Hans-Peter Fuehrer**

Austria

**Francisco-Javier Gamo**

Spain

**Paul Garner**

UK

**Michelle Gatton**

Australia

**Teshome Gebre-Michael**

Ethiopia

**Hellen Gelband**

USA

**Jaline Gerardin**

USA

**Peter Gething**

UK

**Azra Ghani**

UK

**Myriam Gharbi**

France

**Federica Giardina**

Switzerland

**Gabriella Gibson**

UK

**Sabine Gies**

Burkina Faso

**Jose Pedro Gil**

Sweden

**Stresman Gillian**

UK

**Paul Gilson**

Australia

**John Gimnig**

USA

**Emanuele Giorgi**

UK

**Romain Girod**

French Guiana

**Sophie Githinji**

Kenya

**Suchi Goel**

Sweden

**Daniel Goldberg**

USA

**Iveth Gonzalez**

Switzerland

**Raquel Gonzalez**

Spain

**Iveth Jimena González Jiménez**

Switzerland

**Lilia Gonzalez-Ceron**

Mexico

**Louis Clement Gouagna**

Reunion

**Mark Grabowsky**

USA

**Francesco Grandesso**

France

**Patricia Graves**

Australia

**Justin Green**

UK

**Michael Green**

USA

**Bryan Greenhouse**

USA

**Brian Greenwood**

UK

**Jamie Griffin**

UK

**Ulla Griffiths**

UK

**Martin Peter Grobusch**

Netherlands

**Charlotte Gryseels**

Belgium

**Philippe Guerin**

UK

**Sin Yee Gun**

Singapore

**Nilupa Gunaratna**

USA

**Nayana Gunathilaka**

Sri Lanka

**Sunetra Gupta**

UK

**Snehalata Gupte**

India

**Julie Gutman**

USA

**Philippe Guyant**

Papua New Guinea

**Prudence Hamade**

UK

**Busiku Hamainza**

Zambia

**Thomas Hanscheid**

Portugal

**Nick Harding**

UK

**Andrew Hardy**

UK

**Steven Harvey**

USA

**Ahmed Hassanali**

Kenya

**Susanna Hausmann**

Switzerland

**Michael Hawkes**

Canada

**Bill Hawley**

Indonesia

**Michael Hegg**

USA

**Casper Hempel**

Denmark

**Socrates Herrera**

Colombia

**Jim Herrington**

USA

**Manuel Hetzel**

Switzerland

**Matthew Higgins**

UK

**Jenny Hill**

UK

**Kenji Hirayama**

Japan

**Alexandra Hiscox**

Netherlands

**May Ho**

Canada

**Eva Maria Hodel**

UK

**Richard Höglund**

Thailand

**Anthony Holder**

UK

**Michael Hollingdale**

USA

**Yeonchul Hong**

Republic of Korea

**Toshihiro Horii**

Japan

**Rosalind Howes**

UK

**Wenbiao Hu**

Australia

**Austin Hughes**

USA

**Elizabeth Hunsperger**

USA

**Hilary Hurd**

UK

**Michaela Huson**

Netherlands

**Robert Hutagalung**

Germany

**Nguyen Tien Huy**

Japan

**Lars Hviid**

Denmark

**Mallika Imwong**

Thailand

**Marie Chantal Ingabire**

Rwanda

**Angulo Barturen Inigo**

Spain

**Seth Irish**

UK

**Deus Ishengoma**

Tanzania

**Akira Ishih**

Japan

**Jan Jacobs**

Belgium

**Jerry Jacobson**

USA

**Prasanna Jagannathan**

USA

**Eric James**

USA

**Lubin Jiang**

China

**Masamine Jimba**

Japan

**Jacob Johnson**

USA

**Christopher Jones**

UK

**Somchai Jongwutiwes**

Thailand

**Irina Tatiana Jovel**

Sweden

**Jonathan Juliano**

USA

**Vincent Jullien**

France

**Patrick Kachur**

USA

**Björn Kafsack**

USA

**Edwin Kamau**

Kenya

**Akira Kaneko**

Sweden

**Osamu Kaneko**

Japan

**Almamy Kanté**

USA

**Simon Kariuki**

Kenya

**Stephan Karl**

Australia

**Ronnie Kasirye**

Uganda

**Nuzhat Kaushal**

India

**Kassoum Kayentao**

Mali

**James Kazura**

USA

**Joseph Keating**

USA

**Louise Kelly-Hope**

UK

**Karen Kerkhof**

Belgium

**Jamal Khalife**

France

**Solomon Kibret**

Ethiopia

**Albert Kilian**

Spain

**Gerry Killeen**

Tanzania

**Hugh Kingston**

Thailand

**Karin Kirchgatter**

Brazil

**Laura Kirkman**

USA

**Barbara Klein**

USA

**Michael Klemba**

USA

**Frank Kloprogge**

Thailand

**Saskia Knillmann**

Germany

**Astrid Knoblauch**

Switzerland

**Tessa Knox**

Switzerland

**Tamaki Kobayashi**

USA

**Kevin Kobylinski**

USA

**Lizette Koekemoer**

South Africa

**Jacob Koella**

Switzerland

**Hannah Koenker**

USA

**Constantianus Koenraadt**

Netherlands

**Cristian Koepfli**

Australia

**Benjamin Koudou**

UK

**Moritz Kraemer**

UK

**Randall Kramer**

USA

**Katharina Kreppel**

UK

**Benno Kreuels**

Germany

**Sabina Krief**

France

**Andreas Kudom**

Ghana

**Sanjai Kumar**

USA

**Kwadwo Kusi**

Ghana

**Daniel Kyabayinze**

Uganda

**Dennis Kyle**

USA

**Paul Labarre**

USA

**Marcus Lacerda**

Brazil

**Alexis Lacue**

USA

**Jean-Christophe Lagier**

France

**Sham Lal**

UK

**David Lalloo**

UK

**Moses Laman**

Australia

**Tracey Lamb**

USA

**Tobias Landmann**

Kenya

**Jean Langhorne**

UK

**Heather Lanthorn**

USA

**Peter Larson**

USA

**Yee Ling Lau**

Malaysia

**Matthew Laurens**

USA

**Catherine Lavazec**

France

**Arnaud Le Menach**

USA

**Karine Gaelle Le Roch**

USA

**Markus Lee**

UK

**Tovi Lehmann**

USA

**Usa Lek-Uthai**

Thailand

**Bertrand Lell**

Germany

**Christian Lengeler**

Switzerland

**Audrey Lenhart**

USA

**Didier Leroy**

Switzerland

**Andres Lescano**

Peru

**Toby Leslie**

UK

**Jelena Levitskaya**

USA

**Xiaosong Li**

China

**Sue Lightman**

UK

**Direk Limmathurotsakul**

Thailand

**Jean Limongi**

Brazil

**Kim Lindblade**

USA

**Jenny Lindh**

Sweden

**Steve Lindsay**

UK

**Bernt Lindtjorn**

Norway

**Jo Lines**

UK

**Megan Littrell**

USA

**Jenny Liu**

USA

**Manuel Llinas**

USA

**Mike Lo**

South Africa

**Dinora Lopes**

Portugal

**Jose-Juan Lopez-Rubio**

France

**Chris Lorenco**

UK

**Camila Lorenz**

Brazil

**Lena Lorenz**

UK

**Thomas Löscher**

Germany

**Andrew Lover**

Vietnam

**Rachel Lowe**

Spain

**Feng Lu**

Republic of Korea

**Yoel Lubell**

Thailand

**Naomi Lucchi**

USA

**Adrian Luty**

France

**Christine Luxemburger**

France

**Dickson Lwetoijera**

Tanzania

**Gareth Lycett**

UK

**Matthew Lynch**

USA

**Margaret Mackinnon**

Australia

**Pascal Maeser**

Switzerland

**Amanda Maestre**

Colombia

**Stephen Magesa**

Tanzania

**Pascal Magnussen**

Denmark

**Rajendra Maharaj**

South Africa

**Mathieu Maheu-Giroux**

UK

**Kathryn Maitland**

UK

**Silas Majambere**

Tanzania

**Michael Makanga**

South Africa

**Pawan Malhotra**

India

**Benoit Malleret**

Singapore

**David Malone**

UK

**Jessica Maltha**

Belgium

**Sylvie Manguin**

France

**Rashid Mansoor**

UK

**Paola Barbosa Marchesini**

Brazil

**Sean Marcsisin**

USA

**Jutta Marfurt**

Australia

**Claudio Romero Farias Marinho**

Brazil

**Vicki Marsh**

Kenya

**Andreas Mårtensson**

Sweden

**Sandra Martinez**

Colombia

**Josué Martínez-De La Puente**

Spain

**Flor Ernestina Martinez-Espinosa**

Brazil

**Freddie Masaninga**

Zambia

**James Mason**

UK

**Richard Maude**

Thailand

**Alfredo Mayor**

Spain

**Leonard Mboera**

Tanzania

**Charles Mbogo**

Kenya

**Anthony Mbonye**

Uganda

**Robert Mccann**

USA

**James Mccarthy**

Australia

**Malcolm Mcconville**

Australia

**Rose Mcgready**

Thailand

**Alastair Mcgregor**

UK

**Brendan Mcmorran**

Australia

**David Mcvicard**

USA

**Kim Medley**

USA

**Didier Menard**

Cambodia

**Kamini Mendis**

USA

**David Menger**

Netherlands

**Joris Menten**

Belgium

**Martin Meremikwu**

Nigeria

**Steve Meshnick**

USA

**Steven Meshnick**

USA

**Jane Messina**

UK

**Janet Midega**

Kenya

**Alemayehu Midekisa**

USA

**John Miller**

Zambia

**Louis Miller**

USA

**Juan Pablo Millet**

Spain

**Danny Milner**

USA

**Omary Minzi**

Tanzania

**Aline Mirandas**

Brazil

**Andrew Mitchell**

Australia

**Kazutoyo Miura**

USA

**Frank Mockenhaupt**

Germany

**Olugbenga Mokuolu**

Nigeria

**Malcolm Edward Molyneux**

Malawi

**Sassy Molyneux**

Kenya

**Wuelton Monteiro**

Brazil

**Brioni Moore**

Australia

**Cathy Moore**

UK

**Sarah Moore**

Switzerland

**Benjamin Mordmueller**

Germany

**Marta Moreno**

USA

**Ulrika Morris**

Sweden

**Andy Morse**

UK

**William Moss**

USA

**Maria Mota**

Portugal

**Jianbing Mu**

USA

**Atis Muehlenbachs**

USA

**Olaf Mueller**

Germany

**Richard Mukabana**

Kenya

**Pie Müller**

Switzerland

**Courtney Murdock**

USA

**Sean Murphy**

USA

**Lise Musset**

French Guiana

**Clifford Mutero**

South Africa

**James Mutunga**

Kenya

**Collins Mweresa**

Kenya

**Kesara Na-Bangchang**

Thailand

**M. Nacher**

French Guiana

**Behzad Nadjm**

Viet Nam

**Cho Naing**

Malaysia

**Joaniter Nankabirwa**

Uganda

**Bryson Ndenga**

Kenya

**Daniel Neafsey**

USA

**Jim Neilson**

UK

**Scott Nelson**

USA

**Françoise Nepveu**

France

**Charles Newton**

UK

**Paul Newton**

Lao People’s Democratic Republic

**Kija Ng’habi**

Tanzania

**Corine Ngufor**

UK

**S. M. Niaz Arifin**

USA

**Emanuele Nicastri**

Italy

**Mark Nichter**

USA

**Mamoru Niikura**

Japan

**J. Niles**

USA

**Harald Noedl**

Austria

**Paulo Afonso Nogueira**

Brazil

**Gregory Noland**

USA

**Abdisalan Noor**

Kenya

**Francois Nosten**

Thailand

**Christian Nsanzabana**

UK

**Elaine Nsoesie**

USA

**Francine Ntoumi**

Congo

**Myat Htut Nyunt**

Myanmar

**James O’donnell**

Ireland

**Javier Ogembo**

USA

**Mary Chiaka Oguike**

UK

**Jun Ohashi**

Japan

**Bernard Okech**

USA

**Lucy Okell**

UK

**Emelda Okiro**

USA

**Fredros Okumu**

Tanzania

**Joseli Oliveira-Ferreira**

Brazil

**Martin Olivier**

Canada

**Wendy O’meara**

USA

**Sheila Ommeh**

Kenya

**Laura O’reilly**

Switzerland

**Pamela Orjuela-Sanchez**

USA

**Thomas Dan Otto**

UK

**Hans Overgaard**

Norway

**Richard Oxborough**

UK

**Luiz Shozo Ozaki**

USA

**Krijn Paaijmans**

USA

**Norma Padilla**

Guatemala

**Franco Pagnoni**

Switzerland

**Kailash Pandey**

India

**Paul Parham**

UK

**Christian Parobek**

USA

**Gabriel Parra-Henao**

Colombia

**Manuel Patarroyo**

Colombia

**J. Patel**

USA

**Christopher Pell**

Netherlands

**Peter Pemberton-Ross**

Switzerland

**Kristina Persson**

Sweden

**Eskild Petersen**

Denmark

**Margaret Phillips**

USA

**Aung Pyae Phyo**

Thailand

**Gabriel Picone**

USA

**Stephane Picot**

France

**Luc Pieters**

Belgium

**Dylan Pillai**

Canada

**Margaret Pinder**

Gambia

**Elena Pirogova**

Australia

**Richard Pleass**

UK

**Katherine Plewes**

Thailand

**Alexander Ploss**

USA

**Mateusz Plucinski**

USA

**Jeanne Rini Poespoprodjo**

Indonesia

**Mark Polhemus**

Uruguay

**Spencer Polley**

UK

**Marco Pombi**

Italy

**Rodolphe Poupardin**

Czech Republic

**Bruno Pradines**

France

**Ric Price**

Australia

**Judith Prieto**

USA

**Natacha Protopopoff**

UK

**Franck Pugnolle**

France

**Justin Pulford**

Papua New Guinea

**Prasanta Purohit**

India

**Chaturong Putaporntip**

Thailand

**Martha Quinones**

Colombia

**C. P. Raccurt**

Haiti

**Julia Raifman**

USA

**Stuart Alexander Ralph**

Australia

**Michael Ramharter**

Austria

**Milijaona Randrianarivelojosia**

Madagascar

**Pradipsinh Rathod**

USA

**Julian Rayner**

UK

**Michael Reddy**

USA

**Lisa Reimer**

UK

**Robert Reiner**

USA

**Patricia Reis**

Brazil

**Richard Reithinger**

USA

**Eliska Rejmankova**

USA

**E. D. Remarque**

Netherlands

**Laurent Renia**

Singapore

**Dave Richard**

Canada

**Emily Ricotta**

USA

**Michelle Riehle**

USA

**Scott Ritchie**

Australia

**Jacob Riveron**

UK

**Vincent Robert**

France

**Ailie Robinson**

UK

**Kirk Rockett**

UK

**Joacim Rocklöv**

Sweden

**Mario Rodriguez**

Mexico

**Isabel Rodriguez-Barraquer**

USA

**Alfonso Rodriguez-Morales**

Venezuela

**Paul Roepe**

USA

**Meta Roestenberg**

Netherlands

**Stephen Rogerson**

Australia

**Christophe Rogier**

Madagascar

**Elaine Roman**

USA

**Angel Rosas-Aguirre**

Peru

**Philip Rosenthal**

USA

**Amanda Ross**

Switzerland

**Johanna Roth**

Netherlands

**Matthias Rottmann**

Switzerland

**André Rougemont**

Switzerland

**Christian Roussilhon**

France

**Jose Rubio**

Spain

**Daniel Ruiz**

Colombia

**Esmée Ruizendaal**

Netherlands

**Nick Ruktanonchai**

UK

**Tanya Russell**

Australia

**Elizeus Rutebemberwa**

Uganda

**Bernhard Ryffel**

France

**Dinkar Sahal**

India

**Patricia Salgueiro**

Portugal

**Maria Anice Sallum**

Brazil

**Jetsumon Sattabongkot**

Thailand

**Robert Sauerwein**

Netherlands

**David Saunders**

Thailand

**Kim Scarsi**

Canada

**Stephen Schaffner**

USA

**Henk Schallig**

Netherlands

**David Schellenberg**

UK

**Anja Scholzen**

Netherlands

**Birgit Schramm**

France

**Kathrin Schuldt**

Germany

**Ralph Schwarz**

Germany

**Kelly Searle**

USA

**Katarina Selling**

Sweden

**Moh Seng Chang**

Malaysia

**Carlo Severini**

Italy

**Dennis Shanks**

Australia

**Vinod Sharma**

India

**Clive Shiff**

USA

**Samuel Shillcutt**

USA

**Carol Sibley**

USA

**Elisa Sicuri**

Spain

**Christoph Siethoff**

Switzerland

**Sheetal Silal**

South Africa

**Julie Simpson**

Australia

**David Sinclair**

UK

**Neeru Singh**

India

**Marianne Sinka**

UK

**Photini Sinnis**

USA

**Andre Siqueira**

Brazil

**Jacek Skarbinski**

USA

**Ole Skovmand**

France

**Hannah Slater**

UK

**Michael Slotman**

USA

**Larry Slutsker**

USA

**Vincent Sluydts**

Belgium

**Graham Small**

UK

**Renate Corinne Smallegange**

Netherlands

**Jennifer Smith**

USA

**Thomas Smith**

Switzerland

**David Smith**

USA

**Cara Smith Gueye**

USA

**Lucy Smith Paintain**

UK

**Georges Snounou**

France

**Niko Speybroeck**

Belgium

**Michele Spring**

Thailand

**C. N. Srivastava**

India

**Sarah Staedke**

Uganda

**Danielle Stanisic**

Australia

**Peter Starzengruber**

Austria

**Laura Steinhardt**

USA

**Kasia Stepniewska**

UK

**Eleanore Sternberg**

USA

**Jennifer Stevenson**

Zambia

**Justin Stoler**

USA

**Christopher Stone**

Switzerland

**William Stone**

Netherlands

**Luciane Storti-Melo**

Brazil

**Jose Stoute**

USA

**Gro Strom**

Norway

**Erin Stuckey**

USA

**Hugh Sturrock**

USA

**David Sullivan**

USA

**Frank Sullivan**

UK

**Radhika Sundararajan**

USA

**Sinnathamby Surendran**

Sri Lanka

**James Sutcliffe**

Canada

**Colin Sutherland**

UK

**Hiroshi Suzuki**

Japan

**Elin Svensson**

Sweden

**Gote Swedberg**

Sweden

**Harry Tagbor**

Ghana

**Rachida Tahar**

France

**Ambrose Talisuna**

Kenya

**Kathrine Tan**

USA

**Geoff Targett**

UK

**Joel Tarning**

Thailand

**Andrew Tatem**

UK

**Wrj Taylor**

Thailand

**Steve Taylor**

USA

**Terrie Taylor**

USA

**David Tchouassi**

Kenya

**Tilahun Teklehaymanot**

Ethiopia

**Feiko Ter Kuile**

UK

**Anja Terlouw**

UK

**Michael Theisen**

Denmark

**Matthew Thomas**

USA

**Madeleine Thomson**

USA

**Kamala Thriemer**

Australia

**Julie Thwing**

USA

**James Tibenderana**

Uganda

**Leann Tilley**

Australia

**Niraj Tolia**

USA

**Maria Torcia**

Italy

**Baldwyn Torto**

Kenya

**Sarah Tougher**

UK

**Alexandra Towns**

Netherlands

**Harold Townson**

UK

**Katharine Trenholme**

Australia

**Abhai Tripathi**

USA

**Rupam Tripura**

Thailand

**Marita Troye-Blomberg**

Sweden

**Takafumi Tsuboi**

Japan

**Lucy Tusting**

UK

**V. Udhayakumar**

USA

**Juerg Utzinger**

Switzerland

**Neena Valecha**

India

**Denis Valle**

USA

**Tinde van Andel**

Netherlands

**Wim Van Bortel**

Sweden

**Fons J. R. van de Vijver**

Netherlands

**Erika van den Bogaart**

Netherlands

**Bart Van Den Borne**

Netherlands

**Jef Van den Ende**

Belgium

**Philippe Van den Steen**

Belgium

**Anna Maria Van Eijk**

India

**Jean-Pierre Van Geertruyden**

Belgium

**Cornelis Van Noorden**

Netherlands

**Donelly Van Schalkwyk**

UK

**Wesley Van Voorhis**

USA

**Jodi Vanden Eng**

USA

**Amelie Vanteaux**

Cambodia

**Gissella Vasquez**

Peru

**Ashley Vaughan**

USA

**Charles Vaughan-Williams**

South Africa

**Meera Venkatesan**

USA

**Jonathan Vennerstrom**

USA

**Niels Verhulst**

Netherlands

**Kenneth Vernick**

France

**Andreas Verras**

USA

**Lasse Vestergaard**

Philippines

**Ana Margarida Vigário**

Portugal

**Jandouwe Villinger**

Kenya

**Joseph Vinetz**

USA

**Theodor Visser**

USA

**Amy Vittor**

USA

**Michael Von Fricken**

USA

**Lorenz Von Seidlein**

Thailand

**Raj Vuppalanchi**

USA

**Jessica Waite**

USA

**Larry Walker**

USA

**Dorothy Wallace**

USA

**Lance Waller**

USA

**William Walton**

USA

**Ru-Bo Wang**

China

**Zenglei Wang**

USA

**Nicola Wardrop**

UK

**David Waterson**

UK

**David Weetman**

UK

**Walter Weiss**

USA

**Thomas Weitzel**

Chile

**Timothy Wells**

Switzerland

**Edward Wenger**

USA

**Amy Wesolowski**

USA

**Michael White**

UK

**Nicholas John White**

Thailand

**Maxine Whittaker**

Australia

**Christopher Whitty**

UK

**Ryan Wiegand**

USA

**Kyle John Wilby**

Qatar

**Merlin Willcox**

UK

**Craig Williams**

Australia

**Holly Ann Williams**

USA

**Thomas Williams**

Kenya

**Anne Wilson**

UK

**Danny Wilson**

Australia

**Mark Wilson**

USA

**Markus Winterberg**

Thailand

**Sergio Wittlin**

Switzerland

**Michael Witty**

UK

**Charles Wondji**

UK

**Charles Woodrow**

Thailand

**Eve Worrall**

UK

**Colin Wright**

UK

**Blair J. Wylie**

USA

**Rui-De Xue**

USA

**Prashant Yadav**

Spain

**Laith Yakob**

UK

**Stephanie Yanow**

Canada

**Tsin Yeo**

Australia

**Shunmay Yeung**

UK

**Delenasaw Yewhalaw**

Ethiopia

**Stanley Yoder**

USA

**Park Yong-Lak**

USA

**Joshua Yukich**

USA

**Yongyuth Yuthavong**

Thailand

**Sedigheh Zakeri**

Iran

**Fidel Zavala**

USA

**Jingyang Zhang**

USA

**Shuisen Zhou**

China

**Xiao-Nong Zhou**

China

**Charlotte Zikusooka**

South Africa

**Thomas Zoller**

Germany

**Caroline Zöllner**

Germany

**Dejan Zurovac**

Kenya

**Julien Zwang**

Switzerland

